# Physiological Disturbance in Fatty Liver Energy Metabolism Converges on IGFBP2 Abundance and Regulation in Mice and Men

**DOI:** 10.3390/ijms21114144

**Published:** 2020-06-10

**Authors:** Pia Fahlbusch, Birgit Knebel, Tina Hörbelt, David Monteiro Barbosa, Aleksandra Nikolic, Sylvia Jacob, Hadi Al-Hasani, Frederique Van de Velde, Yves Van Nieuwenhove, Dirk Müller-Wieland, Bruno Lapauw, D. Margriet Ouwens, Jorg Kotzka

**Affiliations:** 1 Institute of Clinical Biochemistry and Pathobiochemistry, German Diabetes Center (DDZ), Leibniz Center for Diabetes Research at the Heinrich-Heine-University Düsseldorf, Auf’m Hennekamp 65, 40225 Düsseldorf, Germany; Pia.Fahlbusch@ddz.de (P.F.); Birgit.Knebel@ddz.de (B.K.); Tina.Hoerbelt@uk-essen.de (T.H.); David.Barbosa@hhu.de (D.M.B.); Aleksandra.Nikolic@ddz.de (A.N.); Sylvia.Jacob@ddz.de (S.J.); Hadi.Al-Hasani@ddz.de (H.A.-H.); Margriet.Ouwens@ddz.de (D.M.O.); 2 German Center for Diabetes Research (DZD), 85764 München-Neuherberg, Germany; 3 Institute for Clinical Biochemistry and Pathobiochemistry, German Diabetes Center (DDZ), Medical Faculty, Heinrich Heine University, 40225 Düsseldorf, Germany; 4 Department of Endocrinology, Ghent University Hospital, 9000 Ghent, Belgium; Frederique.VandeVelde@ugent.be (F.V.d.V.); Bruno.Lapauw@uzgent.be (B.L.); 5 Department of Gastrointestinal Surgery, Ghent University Hospital, 9000 Ghent, Belgium; Yves.Nieuwenhove@ugent.be; 6 Clinical Research Centre, Department of Internal Medicine I, University Hospital Aachen, 52074 Aachen, Germany; dirmueller@ukaachen.de

**Keywords:** NAFLD, fatty liver metabolism, fatty liver progression, de novo lipogenesis, IGFBP2, IGF system, methylation, SREBP-1c

## Abstract

Fatty liver occurs from simple steatosis with accumulated hepatic lipids and hepatic insulin resistance to severe steatohepatitis, with aggravated lipid accumulation and systemic insulin resistance, but this progression is still poorly understood. Analyses of hepatic gene expression patterns from alb-SREBP-1c mice with moderate, or aP2-SREBP-1c mice with aggravated, hepatic lipid accumulation revealed IGFBP2 as key nodal molecule differing between moderate and aggravated fatty liver. Reduced IGFBP2 expression in aggravated fatty liver was paralleled with promoter hypermethylation, reduced hepatic IGFBP2 secretion and IGFBP2 circulating in plasma. Physiologically, the decrease of IGFBP2 was accompanied with reduced fatty acid oxidation and increased de novo lipogenesis potentially mediated by IGF1 in primary hepatocytes. Furthermore, methyltransferase and sirtuin activities were enhanced. In humans, IGFBP2 serum concentration was lower in obese men with non-alcoholic fatty liver disease (NAFLD) and steatohepatitis (NASH) compared to non-obese controls, and liver fat reduction by weight-loss intervention correlated with an increase of IGFBP2 serum levels. In conclusion, hepatic IGFBP2 abundance correlates to its circulating level and is related to hepatic energy metabolism and de novo lipogenesis. This designates IGFBP2 as non-invasive biomarker for fatty liver disease progression and might further provide an additional variable for risk prediction for pathogenesis of fatty liver in diabetes subtype clusters.

## 1. Introduction

Non-alcoholic fatty liver disease (NAFLD) covers a broad range of liver disease from simple steatosis to steatohepatitis (NASH) to fibrosis or even cirrhosis. Today, the nature of the disease is supposed to be multifactorial including not only the accumulation of hepatic lipids but also accumulation of toxic lipid or reactive species, insulin resistance, or inflammation [1,2]. In early stages, the accumulation of hepatic lipids is often associated with specific hepatic insulin resistance, while in aggravated lipid accumulation, systemic insulin resistance occurs. Accumulating lipids are most likely derived from increased hepatic lipid production, increased uptake from adipose tissue lipolysis, or diet [3,4,5].

In physiology, the crosstalk between insulin-sensitive tissues defines a finely coordinated network to maintain systemic metabolism. Excess amounts of circulating lipids, as present in obesity, lead to adipose tissue dysfunction, which results in ectopic accumulation of excess lipids in peripheral organs like the liver [6,7]. The increasing amounts of hepatic lipids cause lipotoxicity-mediated changes in hepatic metabolism, which consequently lead to hepatic or even systemic insulin resistance [6,7]. Although the pathogenesis of NAFLD is extensively studied, it remains still unclear which mechanisms lead to the onset and progression of simple steatosis or more severe forms of the disease.

Recently, data-driven analysis clustered patients with diabetes to distinct heterogeneous subtypes to improve risk prediction for associated diseases in individual patients [8]. One of the identified clusters showed a close relation between insulin resistance and the accumulation of hepatic lipids and, consequently, an increased risk for progression of fatty liver disease [8,9]. In addition, several studies assign genetic predisposition to the pathogenesis of fatty liver disease and associated complications [10]. Genetic studies clearly help to understand the pathogenesis of NAFLD and associated complications, but these association studies often lack translation to functional levels to assess physiological and pathological consequences.

In order to identify drivers of NAFLD pathogenesis, our study aimed to identify gene regulatory networks, which determine progression of hepatic lipid accumulation to the point of no return. Here, we used two mouse models with tissue-specific overexpression of the sterol regulatory element-binding protein (SREBP)-1c to discriminate genetic fatty liver mainly derived from increased hepatic de novo lipogenesis and metabolic fatty liver caused by excessive fatty acid influx and ectopic lipid accumulation [11,12].

The transcription factor SREBP-1c is a central regulator of genes involved in lipid and cholesterol synthesis. SREBP-1c is the predominant isoform in lipid metabolism, especially in the activation of de novo lipogenesis (DNL) [13,14]. The regulation of SREBP-1c proteins is complex [15,16,17]. They are regulated on the transcriptional level, by a coordinated proteolytic release of the transcriptional active domain from a precursor molecule, and post-translational modification to regulate transcriptional activity and stability. Each step of this orchestrated regulation integrates information about the metabolic status of a cell into the transactivation of SREBP-1c. The tissue-specific overexpression of the N-terminal transcriptionally active domain of human SREBP-1c circumvents the complex regulation.

The liver-specific transgenic mouse model with SREBP-1c under control of the albumin promoter shows a mild fatty liver, hepatic insulin resistance with compensatory increased β-cell function, and massive obesity, but no signs of inflammation, metabolically healthy adipose tissue, and specific activation of hepatic DNL [11,12].

The second mouse model with adipose tissue specific overexpression of the human transcription factor SREBP-1c included in the study displays a phenotype of fatty liver caused by dramatic increase of systemic lipid load due to the absence of adipose tissue. In these animals, the mechanism responsible for accumulation of lipids in the liver is certainly indirect, as the transgene is not expressed in liver tissue and systemic lipid overflow in the circulation is caused by absent adipose tissue [18].

In sum, in our models the genetic phenotype displays a moderate accumulation of hepatic lipids with specific hepatic insulin resistance, while in the metabolic phenotype, aggravated fatty liver is present with systemic insulin resistance [11,12].

In this study, we applied a transcriptome-wide differential gene expression analysis in liver tissue to identify regulator networks altered between moderate and aggravated fatty liver. Further, we independently validated our findings in primary hepatocytes, as the metabolically active unit of the organ from the different pathologies, and we further analyzed the functional impact on hepatic metabolism. Results derived from mouse studies were translated to men by measurement of the identified candidate, namely insulin-like growth factor binding protein (IGFBP) 2, in sera of obese patients with and without diabetes and biopsy-proven hepatic steatosis (NAFL) or steatohepatitis (NASH), and we further assessed whether intervention that mitigates hepatic steatosis reverses the observed relations.

## 2. Results

### 2.1. Analysis of Regulator Networks in Fatty Liver Pathology

To elucidate the mechanisms involved in the progression of fatty liver diseases, we analyzed metabolically healthy C57Bl6 mice and mouse models with the fatty liver phenotype and insulin resistance, namely the alb-SREBP-1c and the aP2-SREBP-1c models. The alb-SREBP-1c mice introduced to the study had a higher body weight (36.1 g) and a 2.2-fold higher liver fat content (3.37% of liver weight) versus C57Bl6 mice (body weight: 31.7 g, liver fat content: 1.51% of liver weight) (Supplementary Table S1). Furthermore, the alb-SREBP-1c mice displayed hepatic insulin resistance [11] and had higher levels of plasma insulin, leptin, triglycerides, and free fatty acids versus C57Bl6 mice. The aP2-SREBP-1c mice had higher body weight (39.1 g), markedly increased liver fat content (5.35% of liver weight), and according to lipodystrophic phenotype, almost undetectable plasma leptin levels versus the C57Bl6 and alb-SREBP-1c mice. Moreover, the aP2-SREBP-1c mice were characterized by systemic insulin resistance and massive hepatic steatosis [11,18]. Compared to C57Bl6 and alb-SREBP-1c mice, the aP2-SREBP-1c had higher levels of circulating glucose, insulin, triglycerides, and free fatty acids, as well as the liver enzymes ALT, AST, and GLDH (Supplementary Table S1).

**Figure 1:**
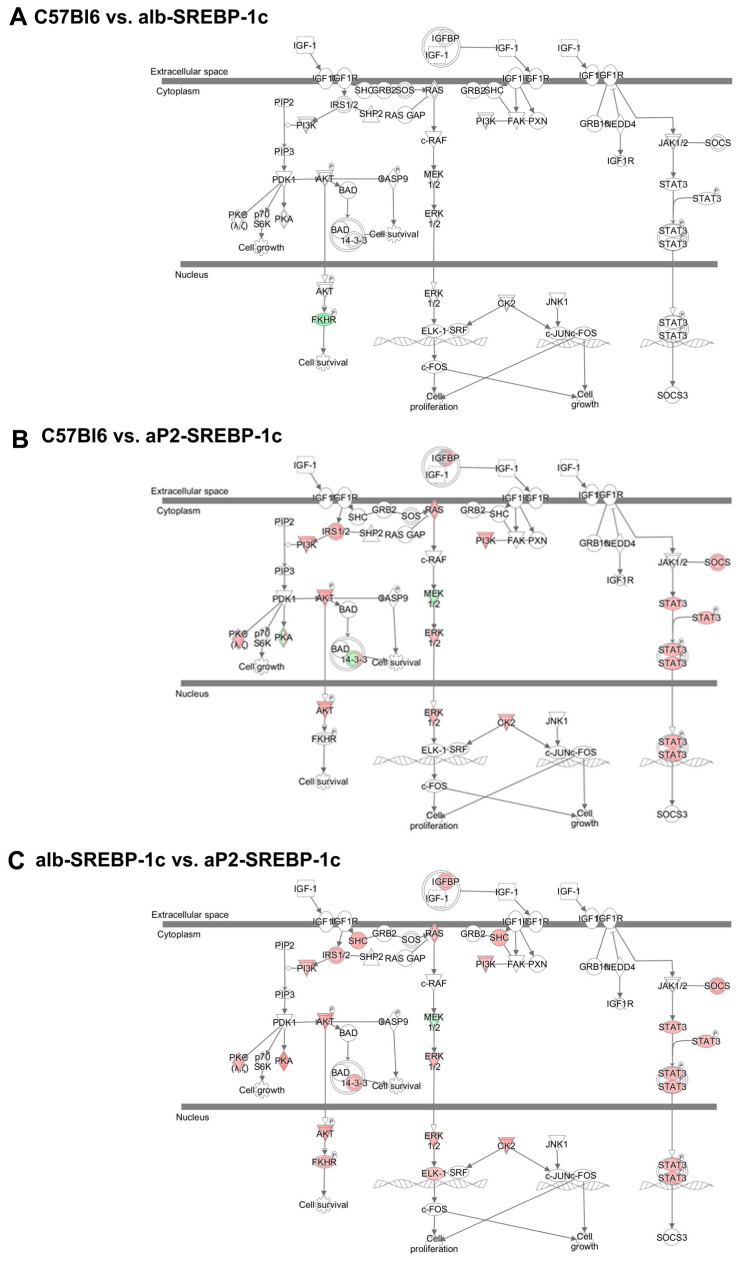
Differential activation of downstream molecules of the insulin-like growth factor (IGF) signaling network. Genes with differential gene expression (1.5-fold, *p*-value < 0.05) were used for IPA^®^ core analyses. Different abundance of genes in comparison to hepatic gene expression of (**A**) C57Bl6 vs. alb-SREBP-1c, (**B**) C57Bl6 vs. aP2-SREBP-1c, and (**C**) alb-SREBP-1c vs. aP2-SREBP-1c liver tissue was overlaid to the canonical IGF signaling pathway. Color code: red—increase in C57Bl6 (**A**,**B**) and alb-SREBP-1c (**C**), green—decrease in C57Bl6 (**A**,**B**) and alb-SREBP-1c (**C**) based on measured expression differences.

In order to identify molecular networks involved in pathology and progression of fatty liver, liver biopsies from the presented mouse models were subjected to transcriptome analyses. The most consistent regulation of hepatic genes in the comparison of alb-SREBP-1c and aP2-SREBP-1c was annotated to the Insulin-like Growth Factor Binding Protein (IGFBP) 2 (consistency score 10.436) using the knowledge-based Ingenuity Pathway Analysis (IPA)^®^ database (Supplementary Figure S1). The IGFBP2 regulator effect is mediated by the specifically reduced abundance of the *Igfbp2* gene product in aP2-SREBP-1c mice and an overall differential regulation of components of the canonical signaling pathway of insulin-like growth factor (IGF), as depicted in Figure 1. The IGF system includes several IGF binding proteins that regulate the bioavailability, transportation, or localization of the growth factors IGF1 and IGF2. The *Igf1* gene is well-conserved among species and is known to be involved in the control of hepatic carbohydrate and lipid metabolism, cell-cycle progression, proliferation and hepatocyte differentiation, hormonal metabolic effects via endo-, auto-, or paracrine regulations, as well as disease pathogenesis [19]. In this study, IGFBP2 was identified as the central effector in knowledge-based gene pathway analysis with only one downstream signaling gene differentially regulated between C57Bl6 versus alb-SREBP-1c, whereas in the aP2-SREBP-1c liver compared to C57Bl6, and even more pronounced if compared to the alb-SREBP-1c liver, a high number of IGF canonical pathway genes showed differential abundance (Figure 1).

**Figure 2:**
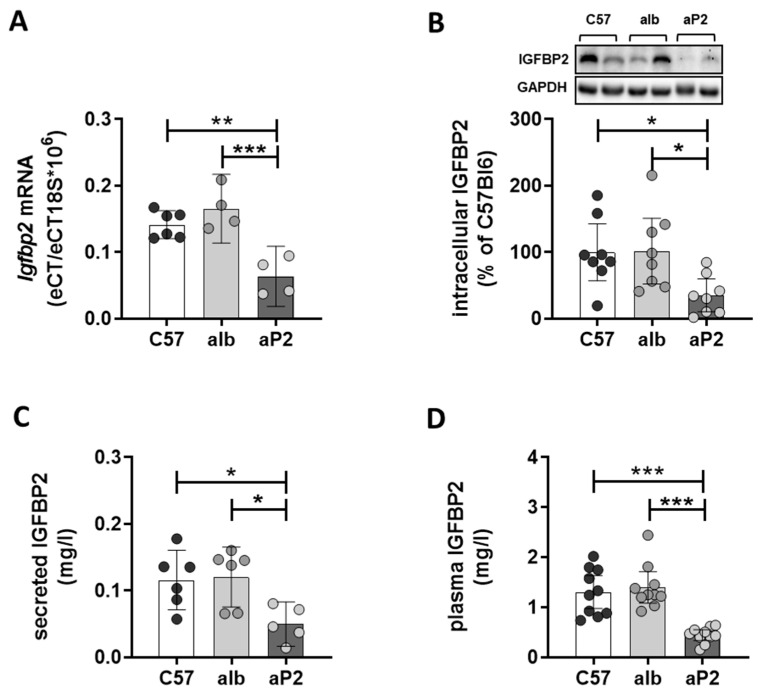
IGFBP2 levels in mouse models. (**A**) *Igfbp2* mRNA expression, (**B**) intracellular IGFBP2 protein content, (**C**) IGFBP2 secretion from primary hepatocytes, and (**D**) plasma IGFBP2 concentration of C57Bl6 (C57), alb-SREBP-1c (alb), and aP2-SREBP-1c (aP2) mice. All data are expressed as mean (±95% CI; *n* = 4–10). Statistics: ANOVA with Sidak correction: *** *p* < 0.001, ** *p* < 0.01 and * *p* < 0.05.

Further investigation focused on the analysis of primary hepatocytes as this cell type represents the metabolically active units in liver tissue and, therefore, allow to link changes in gene expression to disturbances in hepatic metabolism during disease progression. The reduction of *Igfbp2* mRNA expression in aP2-SREBP-1c was independently confirmed in primary hepatocytes by expression analysis (Figure 2A), which was accompanied with decreased intracellular IGFBP2 protein abundance (Figure 2B). The secretion of IGFBP2 protein into cell culture supernatant was significantly reduced in the aP2-SREBP-1c phenotype (Figure 2C) and found to be reflected in a significant reduction of circulating IGFBP2 levels in aP2-SREBP-1c animals (Figure 2D). In addition, direct effects of fatty acid exposure on IGFBP2 secretion were investigated in ex vivo experiments using isolated hepatocytes from metabolically healthy C57Bl6 mice. The results show that chronic exposure to the saturated fatty acid palmitate, but not to the unsaturated fatty acid oleate, reduced the levels of secreted IGFBP2 (Supplementary Figure S2A). This reduction was paralleled with the induction of markers for endoplasmic reticulum stress, namely *Chop* and *Bip* by palmitate, but not oleate in fatty acid treated C57Bl6 hepatocytes (Supplementary Figure S2B,C).

**Figure 3:**
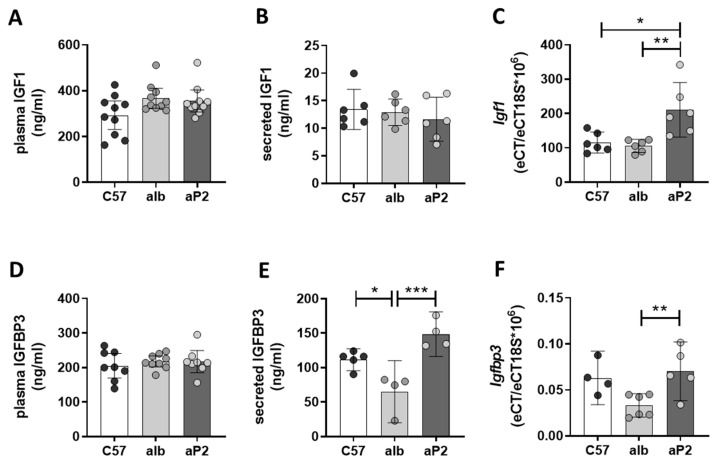
IGF1 and IGFBP3 in mouse models. IGF1 and IGFBP3 levels in plasma (**A**,**D**), culture supernatants (**B**,**E**), and mRNA expression (**C**,**F**) in primary hepatocytes from C57Bl6 (C57), alb-SREBP-1c (alb), and aP2-SREBP-1c (aP2) mice. Data are expressed as mean (±95% CI; *n* = 4–10). Statistics: ANOVA with Sidak correction: *** *p* < 0.001, ** *p* < 0.01 and * *p* < 0.05.

In the circulation, approximately 90% of IGF1 is bound to IGFBP3 [19]. Consequently, IGF1 and IGFBP3 levels were investigated to identify whether reductions of IGFBP2 are accompanied with changes in IGF1 or IGFBP3 abundance to compensate for the loss of IGFBP2 in the aP2-SREBP-1c fatty liver phenotype. There were no significant differences of IGF1 and IGFBP3 plasma concentrations among the phenotypes (Figure 3A,D). IGF1 secretion showed no differences between the analyzed phenotypes (Figure 3B), but a significantly higher *Igf1* mRNA expression was observed in the aP2-SREBP-1c phenotype (Figure 3C). IGFBP3 was secreted in significantly lower concentrations from alb-SREBP-1c compared to C57Bl6 and aP2-SREBP-1c hepatocytes (Figure 3E). *Igfbp3* mRNA expression was reduced in alb-SREBP-1c hepatocytes compared to aP2-SREBP-1c primary hepatocytes, while it remained unchanged between C57Bl6 and aP2-SREBP-1c (Figure 3F).

**Figure 4:**
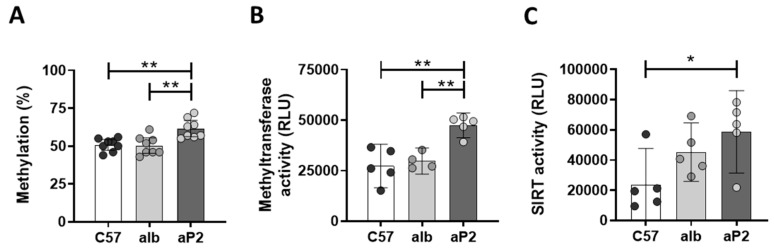
DNA methylation in isolated hepatocytes. (**A**) Methylation state of the mouse *Igfbp2* promoter (position 2912, sequence ID: AC121498.12), (**B**) activity of methyltransferases, and (**C**) sirtuins (SIRT) in hepatocyte whole-cell lysates from C57Bl6 (C57), alb-SREBP-1c (alb), and aP2-SREBP-1c (aP2) mice. Data are expressed as mean (±95% CI; *n* = 4–8). Statistics: ANOVA with Sidak correction: ** *p* < 0.01 and * *p* < 0.05. RLU: relative luminescence units.

Previous studies showed that I*gfbp2* gene expression is potentially regulated via promoter hypermethylation [20,21]. Hence, the *Igfbp2* promoter region was analyzed to elucidate whether reduced *Igfbp2* mRNA expression in aP2-SREBP-1c mice is accompanied with increased cytosine methylation. C57Bl6 and alb-SREBP-1c hepatocyte DNA showed no differences in methylation of the analyzed Igfbp2 promoter region with approximately 50% methylcytosines respectively, while hypermethylation was found specifically in aP2-SREBP-1c hepatocyte DNA with 61% methylated cytosines (Figure 4A). The transfer of methyl groups to DNA is catalyzed by DNA methyltransferases. In the aP2-SREBP-1c hepatocytes, increased promoter methylation went along with an increase in methyltransferase activity (Figure 4B). Further, the NAD^+^-dependent protein deacetylase sirtuin (SIRT) 1 plays a key role in hepatic glucose, fatty acid and energy metabolism. Consequently, SIRT activity serves as a molecular link between the energy status and the adaptive transcriptional responses of the hepatocyte [22]. The activity of SIRT in whole cell lysates from the investigated phenotypes showed a gradual increase of SIRT activity accompanied with increasing amounts of hepatic lipids from C57Bl6 to alb-SREBP-1c to aP2-SREBP-1c phenotypes (Figure 4C).

### 2.2. IGFBP2 Blunts Hepatic IGF1 Action

**Figure 5:**
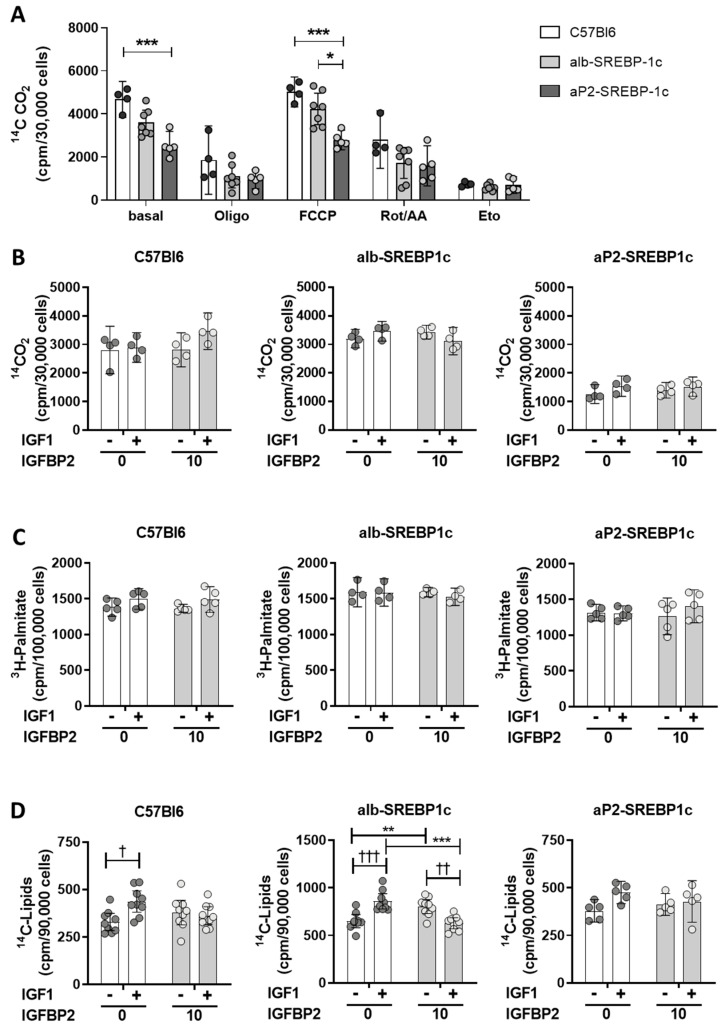
Oxidative capacity and effects of IGF1 and IGFBP2 on lipid metabolism in hepatocytes. (**A**) Hepatocytes from C57Bl6 (C57), alb-SREBP-1c (alb), and aP2-SREBP-1c (aP2) mice were analyzed for palmitate oxidation in untreated cells or under inhibitors of mitochondrial activity (Oligomycin (Oligo), Carbonyl cyanide 4-(trifluoromethoxy)phenylhydrazone (FCCP), Rotenone/Antimycin A (Rot/AA) and Etomoxir (Eto)) (**B**) palmitate oxidation, (**C**) palmitate uptake, and (**D**) de novo lipogenesis under basal conditions and in response to IGF1, IGFBP2, and IGF1 pre-bound to IGFBP2. The bar graphs represent mean (±95% CI; *n* = 4–9). Statistics: ANOVA with Sidak correction: †††, ††, and † indicate *p* < 0.001, *p* < 0.01 and *p* < 0.05 for the effects of IGF1; *** *p* < 0.001, ** *p* < 0.01 and * *p* < 0.05for the illustrated comparisons. Cpm: counts per minute.

IGF growth factors have a central role in hepatic metabolic signaling. Binding of IGF1 to its receptor or the insulin/IGF1 hybrid receptor mediate signaling cascades, e.g., via the mitogen or PI3K-Akt signaling cascades (Figure 1) directly affecting hepatic carbohydrate and lipid metabolism. Hence, the effect of recombinant IGF1 and IGFBP2 was tested in vitro to elucidate IGF1-dependent or -independent effects of recombinant IGFBP2 in hepatocyte fatty acid metabolism. In untreated hepatocytes, the aP2-SREBP-1c phenotype showed the lowest oxidative potential rate of fatty acid oxidation compared to the remaining mouse phenotypes (Figure 5A, basal). Inhibition of mitochondrial electron transport chain either by oligomycin or rotenone and antimycin A reduced oxidation of fatty acids in all phenotypes, while uncoupling of the mitochondrial membrane using carbonyl cyanide 4-(trifluoromethoxy)phenylhydrazone (FCCP) showed a similar pattern as untreated hepatocytes, again with the lowest rate of uncoupled oxidation in aP2-SREBP-1c cells (Figure 5A). Blocking fatty acid transport into mitochondria by etomoxir treatment served as experimental control and indicates the level of non-mitochondrial fatty acid oxidation, which showed no differences in all phenotypes (Figure 5A). The interaction of IGFBP2 with IGF1 signaling showed that neither IGF1 nor IGFBP2 alone, nor the IGF1-IGFBP2 complex, induced changes in fatty acid oxidation (Figure 5B) or fatty acid uptake (Figure 5C) in primary hepatocytes, irrespective of the phenotype. In DNL experiments, IGF1 treatment showed increased DNL in C57Bl6 hepatocytes, but this effect was not observed in cells incubated with the IGF1-IGFBP2 complex, as well as exposing hepatocytes to IGFBP2 alone (Figure 5D). The liver-specific overexpression of SREBP-1c resulted in a high rate of DNL already at the basal level in alb-SREBP-1c hepatocytes (Figure 5D). Nevertheless, both IGF1 and IGFBP2 were found to further enhance DNL in alb-SREBP-1c hepatocytes (Figure 5D). However, when alb-SREBP-1c hepatocytes were incubated with the IGF1-IGFBP2 complex, the rate of DNL remained at basal level. Hepatocytes from aP2-SREBP-1c mice showed a basal rate of DNL at a comparable level to C57Bl6 hepatocytes (Figure 5D). IGF1 incubation enhanced DNL in aP2-SREBP-1c phenotypes, yet this stimulatory effect was not observed when IGF1 was pre-incubated with IGFBP2 (Figure 5D).

### 2.3. IGFBP2 Levels in Humans with Non-Alcoholic Fatty Liver Disease

To examine whether circulating IGFBP2 levels are altered during the course of fatty liver disease progression in humans, we measured IGFBP2 serum concentration in men with class III obesity, with histology confirmed hepatic pathological state (HepObster study) [23]. Patients with liver disease were divided according to histological changes in the liver, defined by the NAFLD activity score (NAS). The NAS integrates the presence and progress of pathological changes in liver tissue in regard to steatosis, lobular inflammation, hepatocellular ballooning, and fibrosis, which allows staging of disease progression [24]. In this study, the patients were divided into groups with NAFL (NAS 1–3) or NASH (NAS 3–7) compared to non-obese men as control group. Obese men with NAFL and NASH had significantly higher BMI (44.3 (41.5–47.1) kg/m^2^ and 40.6 (39.0–42.1) kg/m^2^, respectively) and fatty liver indices (FLI) (99.0 (98.6–99.4) and 98.4 (97.5–99.5), respectively) versus control men without obesity (BMI 25 (23.4–26.6) kg/m^2^, FLI 45.8 (31.6–56.0)) (Supplementary Table S2). HOMA-IR was nearly 3-fold higher in obese men with NAFL and 7-fold higher in NASH compared to the control group. HOMA-IR was more than 2-fold higher in obese men with NASH compared to those with NAFL. The patients with NAFL and NASH had higher fasting levels of insulin, triglycerides, ALT, and γGT, and lower HDL cholesterol as compared to controls. However, no significant differences in these parameters were seen between the NAFL and NASH groups. The participants with NASH had higher levels of fasting glucose and AST, and lower LDL cholesterol versus controls and patients with NAFL (Supplementary Table S2).

**Figure 6:**
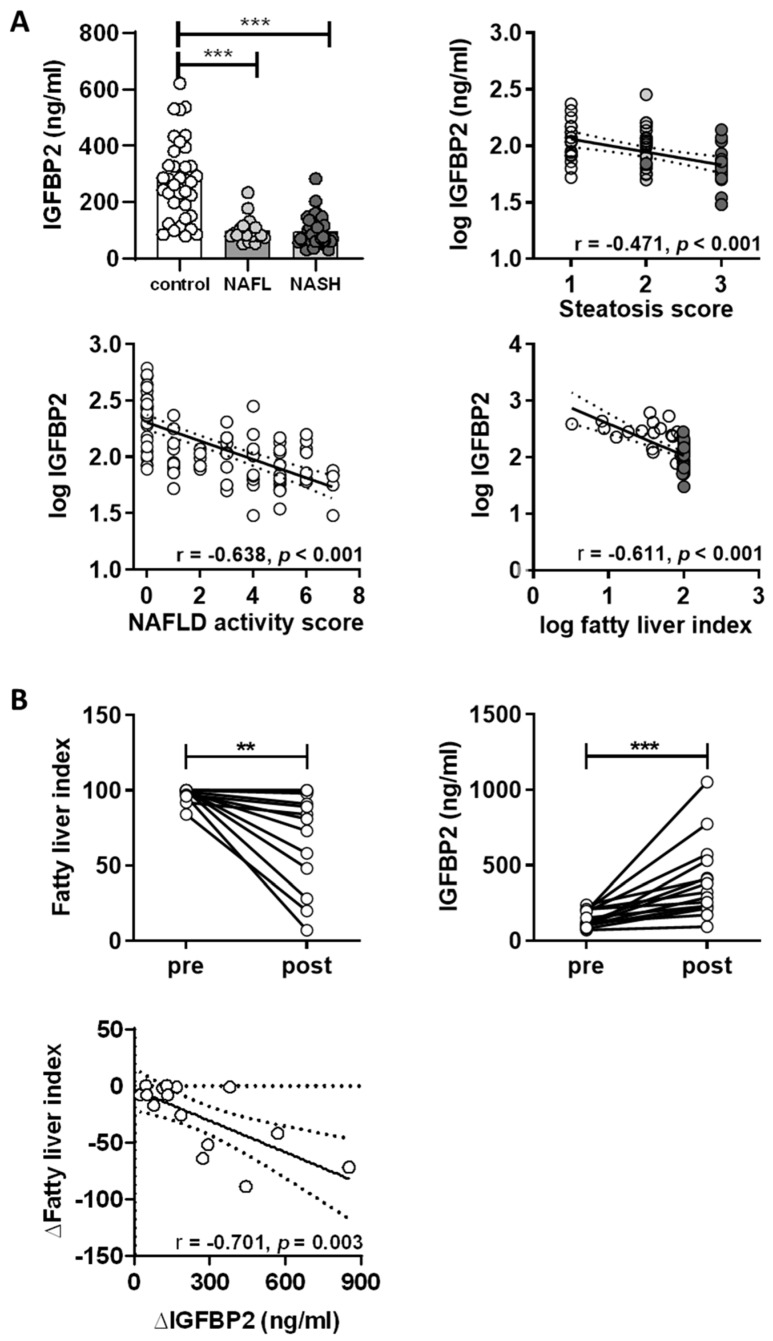
IGFBP2 plasma levels in obese men with NAFLD. (**A**) Circulating IGFBP2 levels in men without obesity (*n* = 36), and biopsy-proven NAFL (*n* = 20) and NASH (*n* = 42), and correlates between IGFBP2 levels with histology steatosis score, NAFLD activity score, and fatty liver index. (**B**) Effect of bariatric intervention on fatty liver index, IGFBP2 serum concentration, and regression analysis for the change (Δ) in fatty liver index versus the change in IGFBP2 levels in class III obese men before (pre) and two years after (post) bariatric surgery. Bar graphs show mean (±95% CI), scatter plots show regression lines (±95% CI, dashed lines). Statistics: ANOVA with Sidak correction. Pearson’s *r* and *p*-values are shown for correlation analysis. ***, ** indicate *p* < 0.001 and *p* < 0.01.

IGFBP2 levels in control men were 279.4 (231.6–327.3) ng/mL versus 99.3 (79.2–119.3) ng/mL and 97.2 (81.6–11.8) ng/mL in subjects with NAFL and NASH, respectively (both *p* < 0.001 versus controls) (Figure 6A). IGFBP2 serum concentrations were not significantly different between obese men with NAFL and NASH. Circulating IGFBP2 levels were associated with the histology-based hepatic steatosis score (*r* = −0.471, *p* < 0.001) (Figure 6A), but not with inflammation, ballooning, or fibrosis scores in the analyzed patients (Supplementary Figure S3). Further, IGFBP2 showed significant association with the NAS (*r* = −0.638, *p* < 0.001) and the calculated FLI (*r* = −0.610, *p* < 0.001) (Figure 6A). IGF1 and IGFBP3 serum concentrations were also assessed in this human cohort to elucidate whether there are compensatory effects for the loss of IGFBP2. In the NAFL and NASH groups, mean IGF1 serum levels were lower compared to control men, while IGFBP3 serum concentrations were not different (Supplementary Figure S3). Pearson analysis showed that IGFBP2 levels associated negatively with BMI (*r* = −0.676, *p* < 0.001), fasting insulin (*r* = −0.343, *p* = 0.001), serum triglycerides (*r* = −0.382, *p* < 0.001), ALT (*r* = −0.467, *p* < 0.001), AST (*r* = −0.277, *p* = 0.006), and γGT (*r* = −0.460, *p* < 0.001), and positively with serum free fatty acids (*r* = 0.226, *p* = 0.043) and HDL cholesterol (*r* = 0.406, *p* < 0.001) (Supplementary Table S3, Supplementary Figure S3). All associations remained significant in a linear regression analysis with adjustment for age. After additional correction for BMI, the associations between IGFBP2 and FLI, triglycerides, ALT, and γGT remained statistically significant (Supplementary Table S4).

### 2.4. Impact of Weight Loss Intervention on Serum IGFBP2 Levels

We examined the effects of gastric bypass surgery on men with class III obesity and severe steatosis (Obster study) [25]. The participants had a BMI of 44.1 (39.7–48.5) kg/m^2^ and a FLI of 97.7 (95.2–100.1) (Supplementary Table S5). Two years after gastric bypass surgery, BMI and FLI were significantly reduced to 34.3 (29.9–38.7) kg/m^2^ and 71.7 (54.0–89.3), respectively (Figure 6B, Supplementary Table S5). The weight loss and reductions in hepatic fat content were further accompanied by reductions in fasting glucose, fasting insulin, triglycerides, ALT, AST, γGT, and by increased HDL cholesterol and improved insulin sensitivity as estimated by HOMA-IR (Supplementary Table S5). In these patients, circulating levels of IGFBP2 increased from 146 (114.8–177.2) to 395 (255.3–534.7) ng/mL, and the change in FLI associated negatively with the change in IGFBP2 levels (*r* = −0.701, *p* = 0.003) (Figure 6B)

## 3. Discussion

This study identifies IGFBP2 as the most consistent effector network of differential gene expression in fatty liver, dependent on the degree of fatty liver. Physiologically this is accompanied by reduced fatty acid oxidation, increased methyltransferase and sirtuin activity, hypermethylation of the *Igfbp2* promoter, and concomitant decreases in *Igfbp2* mRNA and protein abundance. Furthermore, IGFBP2 secretion from primary mouse hepatocytes directly correlated with circulating IGFBP2 plasma levels, which show a reduction depending on the degree of fatty liver. Ex vivo, exposure to the saturated fatty acid palmitate lowers IGFBP2 protein secretion by primary hepatocytes even in metabolically healthy mice. We further show that IGFBP2 blunts the stimulation of de novo lipogenesis by IGF1 in hepatocytes from healthy mice or mouse models with fatty liver disease. Consequently, reductions in IGFBP2 levels by lipids may aggravate the development of fatty liver disease. Finally, we show that the observations obtained in the mouse models are clinically relevant, as the circulating levels of IGFBP2 are lower in patients with NAFL and NASH, and IGFBP2 levels are restored after weight loss following bariatric surgery along with reductions in hepatic fat content.

The role of IGFBP2 in health and disease is still not fully understood. A negative correlation of IGFBP2 with body composition, BMI [26,27,28,29], metabolic syndrome [29,30], type 2 diabetes mellitus [28], or NAFLD [20], and a positive correlation to insulin sensitivity independent to BMI [31] were described. In contrast, IGFBP2 concentrations are high in patients with anorexia nervosa [32].

Studies in mice support these findings. Overexpression of IGFBP2 reduced the predisposition to obesity and improved insulin resistance under normal and high-fat diet in transgenic mouse models such as the ob/ob mouse or diet-induced obesity models [26,28], supporting a rather systemic impact of IGFBP2 in metabolic diseases including obesity and NAFLD. Here, we show that changes in hepatic IGFBP2 secretion were directly reflected in circulating IGFBP2 plasma levels and metabolic effects. In the fatty liver mouse models, an increase in the degree of hepatocyte lipid accumulation resulted in a decline of IGFBP2 secretion. In parallel, fatty acid oxidation is reduced, but hepatocellular SIRT activity is gradually elevated with increasing lipid content. SIRT1 activity depends on the energy level of a cell, and thereof on alterations in energy and lipid metabolism. Metabolic pathologies interfere with SIRT/NAD^+^-systems [33,34] and SIRT activity, as it is tightly regulated by the cellular availability of NAD^+^ [35]. Therefore, SIRT1 is not only a sensor but also a potential regulator of metabolism [36,37]. SIRT1 has been shown to regulate DNA methyltransferase 1 [38], whereas the methyltransferase activity itself is increased in a kind of feedback mechanism to interfere with regulation of rate-limiting enzymes of NAD^+^ metabolism on the transcriptional level [38]. This may be important to maintain liver functionality and insulin sensitivity in response to altered metabolic pressure [39]. In line with this hypothesis, our data show changes in energy metabolism accompanied with increased activity of SIRT1 and methyltransferase activity along with the gradual increase of hepatic lipid content of the mouse models.

The increased activity of methyltransferases further interferes with gene regulation due to alterations in DNA and histone methylation. Igfbp2 promoter methylation in whole blood cells was shown to correlate with an increased type 2 diabetes risk in patients without obesity [40]. As IGFBP2 abundance was already low before the onset of type 2 diabetes in that study [40], it may probably require further physiological alterations. This is supported by a study using a mouse model of diet-induced obesity, where hypermethylation of the Igfbp2 promoter and reduced *Igfbp2* gene expression in early life correlated with the development of fatty liver and impaired glucose metabolism in adolescence [21]. In our study, we demonstrated that hypermethylation of the specific murine Igfbp2 promoter region is not simply dependent on diabetes state, as the alb-SREBP-1c mice already display specific hepatic insulin resistance but no changes in IGFBP2 secretion. Our data indicate regulation of IGFBP2 to be more dependent on the lipid status of the liver, as shown in the aP2-SREBP-1c mice with systemic insulin resistance and aggravated fatty liver.

The liver metabolism of the alb-SREBP-1c mice, driven by the constitutive active transcription factor hSREBP-1c, permanently builds up triglycerides [12], which could be exported to the adipose tissue for storage. In contrast, aP2-SREBP-1c mice display a lipodystrophic phenotype and do not have this deposit to compensate hepatic lipid overflow. Our ex vivo studies indicate that IGFBP2 secretion was significantly altered when hepatocytes were exposed to the saturated fatty acid palmitate as surrogate for saturated free fatty acids, whereas the unsaturated fatty acid oleate does not interfere with IGFBP2 secretion. Palmitate is known to induce ER stress in primary hepatocytes, which contributes to lipotoxicity [41] and, at least in part, to a decrease in IGFBP2 secretion.

Our data indicate that a combination of impaired cellular pathways, regulation of gene expression by hypermethylation, and ER stress lead to altered secretion of IGFBP2. Regulated secretion of IGFBP2 might, therefore, indicate cellular dysfunction of hepatocytes rather than severe cellular damage as indicated by commonly used liver markers, for instance transaminases.

The mechanistic observations drawn from the experimental mouse models might also point towards a function of IGFBP2 as hepatokine. An autocrine loop might interfere with the activation and activity of metabolic sensors like SIRT and methyltransferases to modulate gene expression, including the direct regulation of IGFBP2 expression and consequently the IGF1 effect on metabolic pathways, including DNL.

The translational approach of our investigation supports this hypothesis. In humans, we found lower IGFBP2 serum concentration in obese men with NAFL or NASH compared to control men without obesity. In men with class III obesity, IGFBP2 levels correlated with the grade of hepatic steatosis and disease progression staged by the NAFLD activity score. In addition, recent data published by Wittenbecher et al. showed circulating IGFBP2 serum concentration to be associated with FLI, serum triglyceride, ALT, and γGT levels, as well as a high risk for the development of metabolic complications in patients without obesity [40], further indicating a potential link to IGFBP2 serum concentration and liver function. Even in patients without obesity, a negative association between FLI and IGFBP2 serum concentration is accompanied by an increase of BMI and waist circumference [40] indicating a gradual decrease of IGFBP2 with the degree of fatty liver and adipose tissue function. Furthermore, in the intervention study presented here, in men with obesity the significant reduction of BMI two years after surgery was accompanied with the reduction of fatty liver and serum IGFBP2 concentration. Taken together, the metabolic improvement of adipose tissue by invasive reduction also interfered with the metabolic health of the adipose tissue and improved liver function.

NAFLD often associates with impaired insulin sensitivity and type 2 diabetes, as in the studies presented here. Recently, Ahlqvist et al. found that the heterogeneity of type 2 diabetes can be clustered according to distinct clinical variables to take the individual nature of the disease into consideration [8]. Among these, patients grouped to the severe insulin-resistant diabetes (SIRD) cluster showed low whole-body insulin resistance and the highest hepatic lipid content when newly diagnosed with type 2 diabetes, and in the 5-year follow up analysis they were shown to develop more severe NAFLD [8,9]. In our study, IGFBP2 showed no changes in its serum concentration in terms of specific hepatic insulin resistance, but a significant reduction in serum was related to aggravate liver fat when whole-body insulin resistance is present. In respect to this, IGFBP2 might serve as an additional variable to better classify hepatic status in patients assigned to the SIRD cluster as well as a risk predictor for the development of more severe forms of NAFLD.

The study groups investigated here have some limitations. The data obtained in animal and human data sets are related to male subjects only. Mouse liver tissue in aP2-SREBP-1c showed portal and lobular inflammation without hepatocellular ballooning or fibrosis in histology-based assessment of hepatic pathological changes [11], limiting direct comparison to human steatohepatitis pathology. Liver biopsies from the HepObster control group were not subjected to histological assessment of fatty liver disease state. The absence of liver disease in non-obese control patients was based on overall good health without medication and with normal liver function tests [23]. Human serum analysis was derived from a relatively small number of patients and needs further investigation in larger-scaled studies. Liver biopsy material to verify hepatocyte physiological function, as performed in the animal studies, was not available for use in this study. Furthermore, obesity, type 2 diabetes, and NAFLD share common causative metabolic impairments that affect whole-body metabolism. This implicates caution to differentiate whether the observed association of IGFBP2 with the degree of liver fat is an observation restricted to fatty liver disease. According to study design, FLI was calculated for both human cohorts to strengthen comparability of the results for the degree of fatty liver.

In conclusion, an RNA screening approach for regulatory network genes between mouse models with different degrees of fatty liver identified IGFBP2, a molecule integrated in the IGF system, as the central mediator between moderate and aggravated fatty liver. These results were verified on the mRNA and protein levels, and secretion from primary hepatocytes depends on the degree of fatty liver in mice. Exposure to the saturated fatty acid palmitate is sufficient to lower IGFBP2 secretion in metabolically healthy mice. Mechanistically, IGFBP2 inhibits IGF1 activation on DNL, and the reduction of IGFBP2 may, therefore, aggravate the development of fatty liver disease. In a translational approach, circulating levels of IGFBP2 were lower in obese men with NAFL as well as in those with NASH and were restored after weight loss intervention along with reductions in hepatic fat content. Our results imply IGFBP2 as non-invasive biomarker for the degree of hepatic lipid accumulation when hepatic fat content exceeds a certain level towards disease progression. Further, this study provides IGFBP2 as a variable which might be added to improve reliability of the type 2 diabetes cluster with increased risk to develop severe NAFLD.

## 4. Materials and Methods

### 4.1. Animals

The animal experiments were approved by the Animal Care Committee of the Heinrich-Heine University (Duesseldorf, Germany, Az.84-02.04.2015.A424, 02 April 2015) and performed under the “Principle of laboratory animal care” (NIH publication No. 85-23, revised 1996) and the German law on animal protection. We used male C57Bl6 mice and male transgenic mice with liver- or adipocyte-specific overexpression of transcriptionally active human SREBP-1c (aa 1-436), respectively [11,12,18]. Mice (18 to 24 weeks) were killed by CO_2_ asphyxiation for liver biopsies or isolation of primary hepatocytes. Blood was collected via cardiocentesis and analyzed for lipids, liver enzymes, glucose, and insulin as described [12]. Primary mouse hepatocytes were isolated by a two-step collagenase perfusion protocol as described [11]. Ex vivo experiments were conducted following serum-free overnight culture.

### 4.2. Transcriptome Analyses of Liver Tissue

RNA (150 ng) from snap-frozen liver biopsies was used for genome-wide expression analyses (*n* = 8: C57Bl6, alb-SREBP-1c; *n* = 4: aP2-SREBP-1c) using Mouse Gene 1.0 ST arrays (Thermo Fisher Scientific, Darmstadt, Germany) [42]. Datasets are available under NCBI GEO accession numbers GSE1322981 [43] and GSE139901.

### 4.3. Protein Analysis

IGFBP2 protein abundance was examined in hepatocyte cell lysates by Western blotting [21] using IGFBP2 (Santa Cruz Biotechnology, Heidelberg, Germany) and GAPDH (Cell Signaling Technology, Danvers, MA, USA) according to the manufacturer’s instructions. IGFBP2, IGFBP3, and IGF1 content was measured in unprocessed culture supernatants obtained from 24 h serum-free hepatocyte cultures, as well as in blood plasma using specific mouse Elisa kits (Abcam, Cambridge, UK/R&D systems, Wiesbaden, Germany).

### 4.4. Gene Expression Analysis

Gene expression was examined in total RNA extracted from cultured primary murine hepatocytes by qPCR with gene-specific hybridization probes (*Igfbp2* (Mm00492632), *Igf1* (Mm00439560), *Igfbp3* (Mm01187817), *Chop* (Mm01135937), *Bip* (Mm00517691), and *18S* (4310893E)) (ThermoFisher Scientific, Darmstadt, Germany) as described [44]. We performed the qPCR experiments in triplicate with the cDNA equivalent of 1.7 ng RNA per reaction in a 12.5 µL reaction volume with 2× qPCR mastermix without UNG (Eurogentec, Cologne, Germany); we used a StepOnePlus instrument (Applied Biosystems, Darmstadt, Germany) equipped with a fast-block using the following run methods: 50 °C/2 min, 95 °C/10 min, followed by 40 cycles of 95 °C/15 s, and 60 °C/60 s. When indicated, hepatocytes were incubated for 48 h with BSA-coupled palmitate, BSA-coupled oleate (all 500 μmol/L), or BSA alone.

### 4.5. Methylation Analysis

Genomic DNA of freshly isolated murine hepatocytes was converted with bisulfite (EpiTect FastDNA Bisulfite kit, Qiagen, Hilden, Germany). The *Igfbp2* promoter was amplified by PCR with a biotinylated forward primer: 5′-GAGTTTTTGGGAATAAAGATAAAAGAGT-3′ and reverse primer: 5′-CCCCAAACAACATTTCTCTCT-3′, and sequenced using 5′-AGATAAAAGAGTTAATAGTAA AGT-3′ as sequencing primer (PyroMark Q96 ID, Qiagen, Hilden, Germany). Methyltransferase activity in whole-cell lysates from primary hepatocytes was analyzed using the MTase-Glo^™^ kit (Promega, Mannheim, Germany). All procedures were performed according to the manufacturer’s instructions.

### 4.6. Lipid Metabolism in Hepatocytes

Primary hepatocytes were exposed overnight to 10 nmol/L IGF1, 10 nmol/L IGFBP2, or 10 nmol/L IGF1 pre-bound to 10 nmol/mL IGFBP2. De novo lipogenesis, fatty acid uptake, and fatty acid oxidation were examined as described [11,44]. Analysis of hepatocyte oxidative potential was performed by addition of 0.5 µM oligomycin, 0.5 µM carbonyl cyanide 4-(trifluoromethoxy)phenylhydrazone (FCCP), 0.5 µM rotenone/antimycine A, and 40 µM etomoxir 15 min prior to fatty acid oxidation analyses as described [11,44]. In brief, fatty acid oxidation analyses were performed in an oxidation chamber. Hepatocytes were seeded (3 × 10^4^ cells/well in a 48-well plate) in fatty acid free serum-starvation medium. For each well an absorbent filter paper soaked with 50 µL 1 M NaOH was placed in an adjacent well. Cells were preincubated (15 min) with FCCP or fatty acid β-oxidation inhibitors including oligomyocin, antimycin A/rotenone, or etomoxir. Cells were exposed to 0.3 µCi [^14^C]-palmitic acid, 57 µM fatty acid free BSA, and 9 µM L-carnitin in serum-starvation medium (4 h at 37 °C, 5% CO_2_). HCl (1 M) was added to release ^14^CO_2_, which was trapped in NaOH-soaked filter papers during overnight incubation (16 h, 37 °C, 5% CO_2_) and assessed by liquid scintillation counting. SIRT activity was measured in whole-cell lysates from primary hepatocytes using the SIRT-Glo™ Assay System (Promega, Mannheim, Germany) according to the manufacturer’s instructions.

### 4.7. Study Cohorts

Samples from two independent, previously described clinical studies were analyzed.

Cohort 1 refers to the samples from the HepObster study (registration number: NCT00740194) [23] that was approved by the Ethics Committee of Ghent University Hospital. Patient sera were provided for use in this study to examine 62 obese men with NAFLD, undergoing bariatric surgery, and 36 men without obesity undergoing abdominal surgery for other reasons as control group. Obese men with liver disease were grouped according to NAFLD activity score (NAS) to NAFL (NAS 1–3; *n* = 22) and NASH (NAS 3–7; *n* = 40) group. Type 2 diabetes was present in *n* = 4 NAFL and *n* = 20 NASH participants according to the American Diabetes Association criteria [45]. Overnight fasting blood samples for clinical routine laboratory parameters and liver biopsies for histological assessment of fatty liver disease state were collected as described [23].

Cohort 2 refers to a subset of 15 obese male patients from the Obster Study (B67020084018) with serum samples available for investigation in this study prior to and two years after bariatric intervention [25].

All studies were performed according to the Declaration of Helsinki, and participants gave their written informed consent before the start of the studies.

### 4.8. Analysis of Clinical Samples

Circulating levels of IGFBP2, IGF1, and IGFBP3 were quantitated in serum samples using Quantikine ELISA kits for human IGFBP2, IGF1, and IGFBP3 (R&D systems, Wiesbaden, Germany). The surrogate index for non-alcoholic fatty liver disease was calculated as fatty liver index (FLI) [46]. Insulin sensitivity was calculated as HOMA-IR [47]. Histological analysis of the liver biopsies to determine steatosis, lobular inflammation, ballooning, and fibrosis were performed as described previously [23]. The histological data were used to calculate the NAFLD activity score based on the grade of steatosis, inflammation, and fibrosis score to discriminate between NAFL and NASH [24,48].

### 4.9. Statistical Analysis

Gene expression data were analyzed with Transcriptome Analysis ConsoleTM (v4.01, Applied Biosystems, Darmstadt, Germany) and the core analyses routine of IPA^®^ (Qiagen, Hilden, Germany) as described [42]. Human data were analyzed using SPSS Statistics (version 25.0; IBM, Armonk, NY, USA) and GraphPad Prism (version 8.0, LaJolla, CA, USA). Data are expressed as means (95% CI) unless noted. Differences between two groups were calculated by Student’s *t*-test. In the case of more groups, one- or two-way ANOVA followed by Sidak correction for multiple comparisons was used. Variables with a skewed distribution were log-transformed prior to linear regression analysis with adjustments for age and/or BMI. A *p*-value of <0.05 was considered as statistically significant.

## Supplementary Materials

Supplementary materials can be found at https://www.mdpi.com/1422-0067/21/11/4144/s1.

## Abbreviations

IGF	insulin-like growth factor
IGFBP	insulin-like growth factor binding protein
NAFLD	non-alcoholic fatty liver disease
NAFL	non-alcoholic fatty liver
NASH	non-alcoholic steatohepatitis
SREBP-1c	sterol regulatory element-binding protein 1c
ALT	alanine aminotransferase
AST	aspartate aminotransferase
DNL	de novo lipogenesis
NAS	NAFLD activity score
BMI	body mass index
FLI	fatty liver index
HOMA-IR	homeostasis model assessment of insulin resistance
γGT	gamma-glutamyl transferase
SIRT	sirtuin
