# Supplementary material for: Physiological Disturbance in Fatty Liver Energy Metabolism Converges on IGFBP2 Abundance and Regulation in Mice and Men
Source: Int J Mol Sci. 2020 Jun 10;21(11):4144. doi: 10.3390/ijms21114144 (PMC7312731; doi:10.3390/ijms21114144)
Supplement: Supplementary file 1 [file ijms-21-04144-s001.pdf]

Supplementary Materials

Physiological Disturbance in Fatty Liver Energy Metabolism Converges on IGFBP2 Abundance and Regulation in Mice and Men

Pia Fahlbusch, Birgit Knebel, Tina Hörbelt, David Monteiro Barbosa, Aleksandra Nikolic, Sylvia Jacob, Hadi Al-Hasani, Frederique Van de Velde, Yves Van Nieuwenhove, Dirk Müller-Wieland, Bruno Lapauw, D. Margriet Ouwens and Jorg Kotzka

Table S1. Mouse characteristics.

Variable	C57Bl6	alb-SREBP-1c	aP2-SREBP-1c	*p*
n	8	8	8	
Age, weeks	24	24	24	
Bodyweight, g	31.7 (28.9–34.6)	36.1 (34.9–37.2) **	39.1 (36.6–41.5) ***,#	<0.001
Liver weight, g	1.58 (1.43–1.72)	1.96 (1.84–2.08)	3.81 (3.06–4.57) ***,###	<0.001
Liver weight, % bodyweight	4.96 (4.91–5.73)	5.44 (5.04–5.83)	9.81 (7.85–11.8) ***,###	<0.001
Liver fat, % liver weight	1.51 (1.37–1.65)	3.37 (3.08–3.66) ***	5.35 (4.66–6.05) ***,###	<0.001
Plasma glucose, mg/dL	127 (114–139)	142 (135–149)	233 (213–253) ***,###	<0.001
Plasma insulin, U/L	0.92 (0.69–1.15)	4.13 (2.51–5.74) ***	7.41 (5.98–8.83) ***,###	<0.001
HOMA-IR	0.29 (0.21–0.37)	1.44 (0.89–1.99) **	4.27 (3.34–5.20) ***,###	<0.001
Triglycerides, mmol/L	113 (92.8–133)	255 (211–300) ***	316 (277–354) ***,#	<0.001
Cholesterol, mmol/L	106 (92.6–120)	125 (116–134) *	127 (114–140) *	0.018
Free fatty acids, mmol/L	1.09 (0.88–1.29)	2.50 (2.16–2.83) ***	3.38 (2.64–4.12) ***,###	<0.001
ALT, U/L	30.1 (26.8–33.5)	58.5 (48.9–68.1)	154 (114–195) ***,###	<0.001
AST, U/L	30.3 (21.6–38.9)	60.8 (47.1–74.4)	279 (213–344) ***,###	<0.001
GLDH, U/L	8.03 (6.03–10.0)	14.4 (6.17–22.7)	25.8 (18.0–33.5) ***,#	<0.001

Data are presented as mean (±95% CI). Differences among the groups were calculated by ANOVA followed by Sidak correction for multiple comparisons. ***, **, * indicate $p < 0.001$, $p < 0.01$, and $p < 0.05$ versus C57Bl6 mice, ###, and # indicate $p < 0.001$ and $p < 0.05$ versus alb-SREBP-1c mice. HOMA-IR: Homeostatic model assessment for insulin resistance; ALT: alanine aminotransferase; AST: aspartate aminotransferase; GLDH: glutamate dehydrogenase; SREBP-1c: sterol regulatory element-binding protein-1c.

Table S2. Patient characteristics HepOBSTER cohort. Control men without obesity (Control) and class III obese men with and without type 2 diabetes and NAFLD (NAFL and NASH).

Variable	Control	NAFL	NASH	*p*
n	36	22	40	
Type 2 diabetes, n	0 (0%)	4 (18%)	20 (50%)	
Age, years	46.2 (41.9–50.6)	42.5 (37.0–48.0)	45.9 (42.9–48.9)	0.434
Body mass index, kg/m^2	25.0 (23.4–26.6)	44.3 (41.5–47.1) ***	40.6 (39.0–42.1) ***	<0.001
NAFLD activity score	0	1–3	3–7	
Fatty liver index	45.8 (31.6–56.0)	99.0 (98.6–99.4) ***	98.4 (97.5–99.5) ***	<0.001
Fasting glucose, mmol/L	5.11 (4.74–5.47)	6.09 (4.70–7.48)	6.63 (5.93–7.34) **	0.002
Fasting insulin, pmol/L	36.9 (29.7–44.1)	97.7 (70.5–125) ***	160 (92.8–226) ***	<0.001
HOMA-IR	1.20 (0.95–1.46)	3.41 (1.60–5.21) ***	8.32 (1.12–15.5) ***	<0.001
Free fatty acids, mmol/L	0.70 (0.57–0.83)	0.59 (0.51–0.66)	0.63 (0.57–0.69)	0.263
Triglycerides, mg/dL	142 (118–165)	190 (166–214) *	244 (175–313) ***	<0.001
Cholesterol, mg/dL	181 (170–193)	186 (174–198)	175 (157–192)	0.100
HDL-cholesterol, mg/dL	46.7 (42.2–51.3)	37.4 (32.8–41.9) **	34.3 (29.9–38.6) ***	<0.001
LDL-cholesterol, mg/dL	104 (93.0–114)	105 (91.5–118)	87.9 (78.5–97.3) *,#	0.019
ALT, IU/L	29.4 (22.0–36.9)	44.6 (35.9–53.2) *	53.5 (42.3–64.7) ***	<0.001
AST, IU/L	25.3 (21.6–29.0)	28.0 (23.4–32.6)	37.5 (28.5–46.4) **	0.002
γGT, IU/L	29.3 (22.0–36.6)	44.6 (35.9–53.2) *	53.5 (42.3–64.7) ***	<0.001

Data are presented as mean (±95% CI). Differences among the groups were calculated by ANOVA followed by Sidak correction for multiple comparisons. ***, **, * indicate $p < 0.001$, $p < 0.01$, and $p < 0.05$ versus normal-weight control men; # indicates $p < 0.05$ versus NAFL. ALT: alanine aminotransferase; AST: aspartate aminotransferase; BMI: body mass index; γGT: γ-glutamyltransferase; HDL: high density lipoprotein; HOMA-IR: Homeostatic model assessment for insulin resistance; LDL: low density lipoprotein; NAFL: non-alcoholic fatty liver; NASH: non-alcoholic steatohepatitis.

Table S3. Exploratory Pearson analysis for IGFBP2 serum levels in HepOBSTER cohort.

Variable	IGFBP2
n	98
Age, years	**0.302 (0.002)**
Body mass index	**−0.676 <0.001)**
Fatty liver index	**−0.611 (<0.001)**
Fasting glucose	−0.152 (0.135)
Fasting insulin	**−0.343 (0.001)**
HOMA-IR	**−0.304 (0.006)**
Free fatty acids	**0.226 (0.043)**
Triglycerides	**−0.382 (<0.001)**
Cholesterol	0.033 (0.748)
HDL-cholesterol	**0.406 (<0.001)**
LDL-cholesterol	0.113 (0.284)
ALT	**−0.467 (<0.001)**
AST	**−0.277 (0.006)**
γGT	**−0.460 (<0.001)**

The data are presented as Pearson's *r* (*p*). Variables with a skewed distribution were log-transformed prior to the analysis. ALT: alanine aminotransferase; AST: aspartate aminotransferase; γGT: γ-glutamyltransferase; HDL: high density lipoprotein; HOMA-IR: Homeostatic model assessment for insulin resistance; LDL: low density lipoprotein. Significant parameters are indicated in bold.

Table S4. Multivariate regression analysis for IGFBP2 serum levels in HepOBSTER cohort.

Variable	Not adjusted	Age	Age + BMI
Body mass index	**−0.676 (<0.001)**	**-0.656 (<0.001)**	n.a.
Fatty liver index	**−0.611 (<0.001)**	**−0.568 (<0.001)**	**−0.358 (0.030)**
Fasting insulin	**−0.343 (0.001)**	**−0.345 (<0.001)**	0.069 (0.445)
HOMA-IR	**−0.297 (0.008)**	**−0.262 (0.013)**	0.135 (0.146)
Free fatty acids	**0.226 (0.043)**	0.210 (0.058)	0.158 (0.083)
Triglycerides	**−0.382 (<0.001)**	**−0.402 (<0.001)**	**−0.163 (0.039)**
HDL-cholesterol	**0.406 (<0.001)**	**0.382 (<0.001)**	0.128 (0.110)
ALT	**−0.467 (<0.001)**	**−0.419 (<0.001)**	**−0.201 (0.010)**
AST	**−0.277 (0.006)**	**−0.260 (0.007)**	−0.132 (0.072)
γGT	**−0.460 (<0.001)**	**−0.413 (<0.001)**	**−0.202 (0.011)**

The data are β (p). Variables with a skewed distribution were log-transformed prior to the analysis. ALT: alanine aminotransferase; AST: aspartate aminotransferase; γGT: γ-glutamyltransferase; HDL: high density lipoprotein; HOMA-IR: Homeostatic model assessment for insulin resistance; LDL: low density lipoprotein. Significant parameters are indicated in bold.

Table S5. Intervention study Obster cohort. Obese men prior to (pre) and 2 years after bariatric surgery (post).

Variable	Pre	Post	*p*
n	15	15	
Age, years	51.1 (44.6–57.6)	53.3 (46.9–59.8)	<0.001
Body mass index, kg/m^2	44.1 (39.7–48.5)	34.3 (29.9–38.7)	<0.001
Fatty liver index	97.7 (95.2–100.1)	71.7 (54.0–89.3)	0.005
Fasting glucose, mmol/L	6.45 (5.5–7.4)	5.45 (5.0–5.9)	0.068
Fasting insulin, pmol/L	164.6 (110.1–219.0)	89.6 (45.7–133.6)	0.001
HOMA-IR	7.29 (4.4–1.6)	3.4 (1.6–5.2)	0.003
Triglycerides, mmol/L	149.3 (104.1–194.5)	103.9 (76.2–131.5)	0.037
Cholesterol, mmol/L	164.9 (148.0–181.7)	167.9 (148.1–187.7)	0.710
HDL-cholesterol, mmol/L	42.4 (36.9–47.9)	59.6 (51.6–67.7)	<0.001
LDL-cholesterol, mmol/L	92.1 (77.8–106.5)	86.7 (70.3–103.2)	0.540
ALT, IU/L	64.9 (36.0–93.8)	31.5 (23.3–39.6)	0.028
AST, IU/L	41.7 (28.0–55.4)	28.9 (20.8–37.1)	0.045
γGT, IU/L	44.7 (18.3–71.0)	35.5 (17.1–54.0)	0.511

Data are presented as mean (±95% CI). Differences among the groups were calculated by paired *t*-test. ALT: alanine aminotransferase; AST: aspartate aminotransferase; γGT: γ-glutamyltransferase; HDL: high density lipoprotein; HOMA-IR: Homeostatic model assessment for insulin resistance; LDL: low density lipoprotein.

A C57Bl6 vs. alb-SREBP-1c

Top Regulator Effect Networks			
ID	Regulators	Diseases & Functions	Consistency Score
1	CSF2,CSF3,EPO,IL1B,MYD88,TNF	Endocytosis by eukaryotic cells (+5 more)	17,5
2	IL27,MYD88	Activation of blood cells,Hepatocellular carcinoma (+1 more)	4,536
3	AKT1	Activation of blood cells	-4,919
4	TP53	Quantity of metal ion	-11,023
5	CSF2	Organismal death	-14,496

B C57Bl6 vs. aP2-SREBP-1c

Top Regulator Effect Networks			
ID	Regulators	Diseases & Functions	Consistency Score
1	TFEB	Degeneration of central nervous system (+3 more)	6,718
2	26s Proteasome,SIRT6,TFEB	Biosynthesis of nucleoside triphosphate (+4 more)	5,578
3	26s Proteasome,Akt,SIRT6,TARDBP,TRAP1	Biosynthesis of nucleoside triphosphate (+2 more)	4,131
4	INSR,MAPK9,PCGEM1	Metabolism of membrane lipid derivative (+1 more)	2,593
5	MYC	Regeneration of liver	1,89

C alb-SREBP-1c vs. aP2-SREBP-1c

Top Regulator Effect Networks			
ID	Regulators	Diseases & Functions	Consistency Score
1	IGFBP2	Anemia,Cell viability of tumor cell lines (+5 more)	10,436
2	TFEB	Ataxia,Autophagy,Degeneration of cells (+4 more)	9,071
3	ATF4,CYP3A,EIF4E,FOXO1,HRAS,IFNA2,LGR4,NR0B 2	Cholesterol transport (+4 more)	8,598
4	ATF4,CYP3A,IgG,NR0B2,SYVN1	Ataxia,Cholesterol transport (+7 more)	7,667
5	SIRT6	Cell viability,Dysglycemia,Insulin sensitivity (+3 more)	7,483

Figure S1. Summary of the 5 top score regulator effect networks from transcriptome analysis of C57Bl6, alb-SREBP-1c and aP2-SREBP-1c liver tissue. Data sets were analyzed with knowledge based IPA® software to identify most consistent networks of regulated gene expression found in comparison of C57Bl6 vs. (**A**) alb-SREBP-1c or (**B**) aP2-SREBP-1c as well as (**C**) alb-SREBP-1c vs. aP2-SREBP-1c.

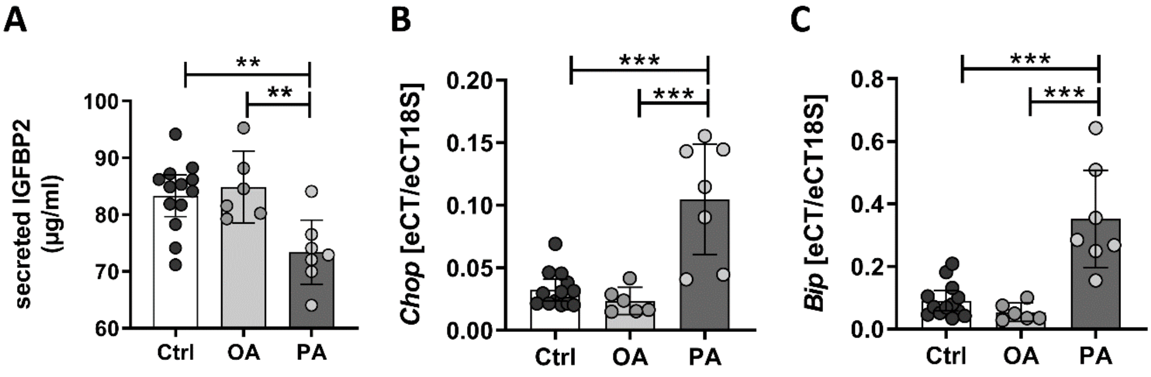

Figure S2. Effects of free fatty acids on metabolically healthy C57Bl6 hepatocytes. (**A**) Concentration of IGFBP2 secretion into culture supernatant from C57Bl6 primary hepatocytes treated with 500 µM BSA-conjugated palmitate (PA), oleate (OA) or the respective amount of BSA alone (Ctrl) in 48 h culture, followed by analysis of (**B**) *Chop* and (**C**) *Bip* mRNA expression. The bar graphs are mean (±95% CI) of 6–7 independent experiments. Statistical differences were calculated by ANOVA followed by Sidak correction for multiple comparisons. **, *** indicate $p < 0.01$ and $p < 0.001$ for labeled comparisons.

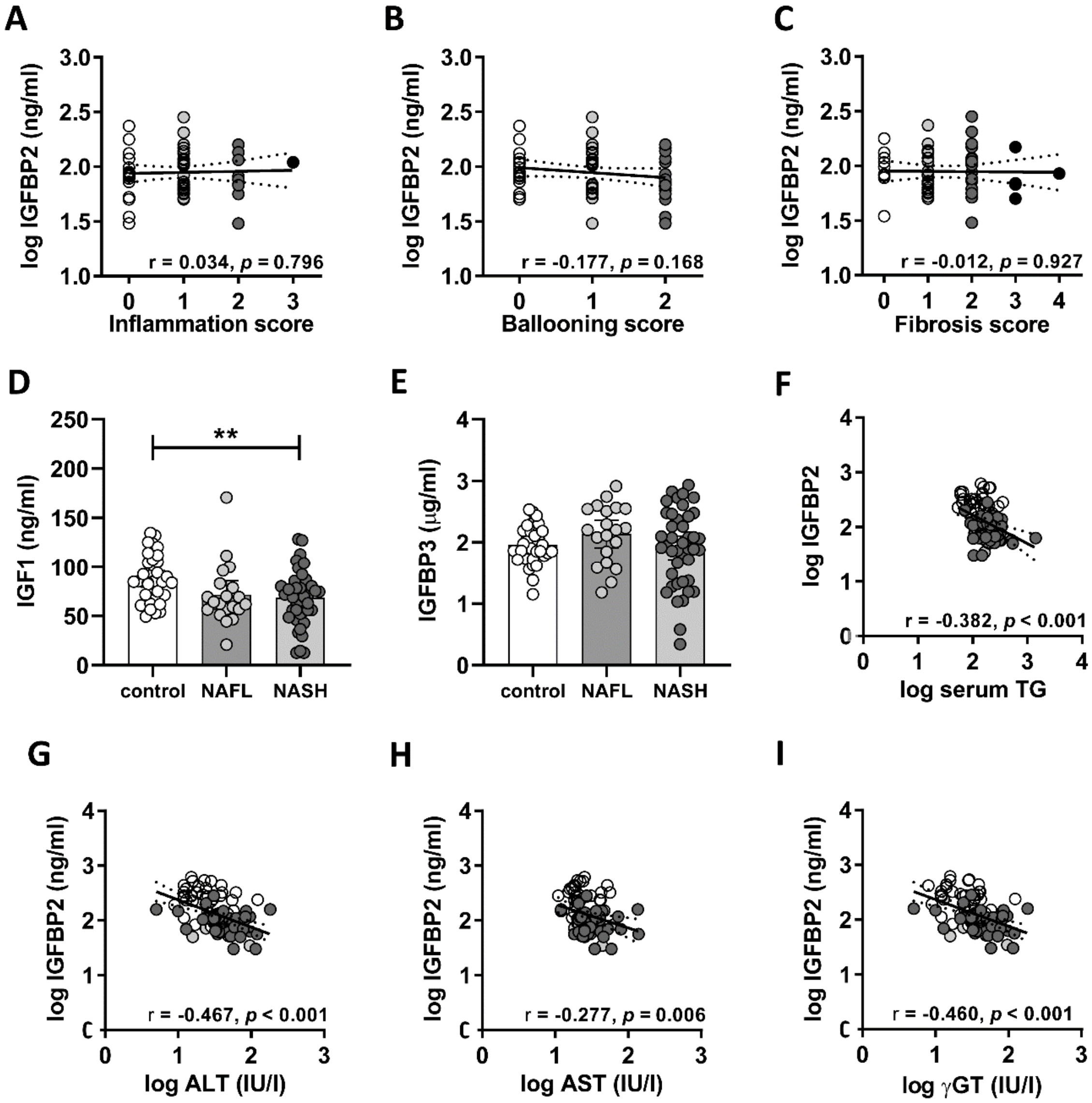

Figure S3. Correlates of circulating IGFBP2 in HepOBSTER cohort. Scatter plots show correlates between circulating IGFBP2 and (**A**) inflammation, (**B**) ballooning and (**C**) fibrosis score, (**F**) serum triglycerides (TG), (**G**) ALT, (**H**) AST, and (**I**) γGT. Bar graphs present circulating (**D**) IGF1 and IGFBP3 (**E**) levels in HepOBSTER cohort. Bar graphs show mean (±95% CI), scatter plots show regression lines (±95% CI, dashed lines). Statistical differences were calculated by ANOVA followed by Sidak correction for multiple comparisons. ** indicate $p < 0.01$ for labeled comparison. Pearson's r and p-values are shown for correlation analysis.
